# Metabolomics Signature in Prediabetes and Diabetes: Insights From Tandem Mass Spectrometry Analysis

**DOI:** 10.1002/edm2.484

**Published:** 2024-05-13

**Authors:** Saad Ayyal Jabbar Al‐Rikabi, Ali Etemadi, Maher Mohammed Morad, Azin Nowrouzi, Ghodarollah Shayriyar Panahi, Mozhgan Mondeali, Mahsa Toorani‐ghazvini, Ensieh Nasli‐Esfahani, Farideh Razi, Fatemeh Bandarian

**Affiliations:** ^1^ Department of Clinical Biochemistry, School of Medicine Tehran University of Medical Sciences Tehran Iran; ^2^ Endocrinology and Metabolism Research Center, Endocrinology and Metabolism Clinical Sciences Institute Tehran University of Medical Sciences Tehran Iran; ^3^ Medical Biotechnology Department, School of Advanced Technologies in Medicine Tehran University of Medical Sciences Tehran Iran; ^4^ Department of Medical Genetics, School of Medicine Tehran University of Medical Sciences Tehran Iran; ^5^ Diabetes Research Center Endocrinology and Metabolism Clinical Sciences Institute, Tehran University of Medical Sciences Tehran Iran; ^6^ Metabolomics and Genomics Research Center Endocrinology and Metabolism Molecular‐Cellular Sciences Institute, Tehran University of Medical Sciences Tehran Iran

**Keywords:** data analysis, diabetes, metabolomics tandem mass spectrometry, noncommunicable diseases

## Abstract

**Objective:**

This study investigates the metabolic differences between normal, prediabetic and diabetic patients with good and poor glycaemic control (GGC and PGC).

**Design:**

In this study, 1102 individuals were included, and 50 metabolites were analysed using tandem mass spectrometry. The diabetes diagnosis and treatment standards of the American Diabetes Association (ADA) were used to classify patients.

**Methods:**

The nearest neighbour method was used to match controls and cases in each group on the basis of age, sex and BMI. Factor analysis was used to reduce the number of variables and find influential underlying factors. Finally, Pearson's correlation coefficient was used to check the correlation between both glucose and HbAc1 as independent factors with binary classes.

**Results:**

Amino acids such as glycine, serine and proline, and acylcarnitines (AcylCs) such as C16 and C18 showed significant differences between the prediabetes and normal groups. Additionally, several metabolites, including C0, C5, C8 and C16, showed significant differences between the diabetes and normal groups. Moreover, the study found that several metabolites significantly differed between the GGC and PGC diabetes groups, such as C2, C6, C10, C16 and C18. The correlation analysis revealed that glucose and HbA1c levels significantly correlated with several metabolites, including glycine, serine and C16, in both the prediabetes and diabetes groups. Additionally, the correlation analysis showed that HbA1c significantly correlated with several metabolites, such as C2, C5 and C18, in the controlled and uncontrolled diabetes groups.

**Conclusions:**

These findings could help identify new biomarkers or underlying markers for the early detection and management of diabetes.

## Graphical abstract

This study investigates the metabolic differences between normal, prediabetic and diabetic patients with good and poor glycaemic control (GGC and PGC). These findings could help identify new biomarkers or underlying markers for the early detection and management of diabetes.

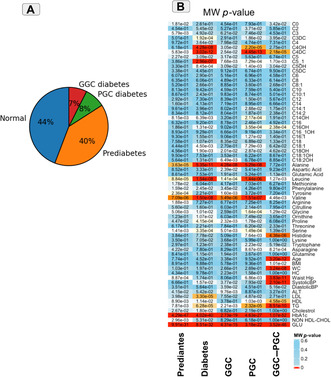

## 1 Introduction

Diabetes mellitus (DM) is a prevalent metabolic disorder resulting from a deficiency in insulin release, insulin action or a combination of both [1]. Hyperglycaemia as the hallmark of diabetes, along with other disturbances in carbohydrate, lipid and protein metabolism, leads to the development of life‐threatening and debilitating complications such as microvascular (neuropathy, nephropathy and retinopathy) and macrovascular (coronary heart disease, cerebrovascular disease and peripheral vascular disease) demonstrations. These complications are responsible for the morbidity and mortality of this disease [2, 3]. Biomarkers, often blood parameters, are used as an indicator of a physiological or pathological process and thus having the potential to predict specific outcomes [4, 5].

Today, metabolomics techniques are widely applied to investigate the metabolic changes in the human body and discover the biomarkers related to disease occurrence [6]. Evidence proposes that aromatic amino acids (AAAs), branched‐chain amino acids (BCAAs) and acylcarnitines (AcylCs) contribute to insulin resistance, showing defects in β‐oxidation, amino acid metabolism and tricarboxylic acid cycle [7]. Despite many studies assessing the metabolite profiles to identify biomarkers of diabetes [8, 9, 10], there is no comprehensive agreement between them that can be attributed to different ethnicities and study designs [5, 11, 12, 13]. Also, the study of patients with DM at different stages of the disease was less accomplished [14]. Therefore, we designed a large case–control study that employs LC–MS/MS‐based metabolomics technique to evaluate plasma amino acid and AcylC metabolites in the prediabetes and diabetes (poor glycaemic control [PGC] and good glycaemic control [GGC]) groups compared with the healthy group.

## 2 Material and Methods

### 2.1 Participants

The initial raw data frame was 1102 people extracted from our previous study of the Surveillance of Risk Factors of NCDs in Iran Study (STEPS 2016) [15], in which participants were randomly selected from Iranian adults. The study subjects underwent a thorough questionnaire, followed by a series of anthropometric measurements. Participants were instructed to fast for 8–12 h prior to blood sampling at the laboratory. Biochemical analysis was performed using Cobas C311 autoanalyzer from Roche company.

The study protocol was approved by the Ethics Committee of Endocrinology and Metabolism Clinical Sciences Institute, Tehran University of Medical Sciences (IR.TUMS.EMRI.REC. 1395.00141) and performed under the Declaration of Helsinki. The purpose of the study was explained to the participants, and written informed consent was obtained from all participants.

### 2.2 Tandem Mass Spectrometry

We utilised a standard HPLC system (Thermo Scientific Dionex UltiMate 3000) with a triple quadrupole mass spectrometer API 3200 (SCIEX) operating in positive electrospray ionisation mode to perform MS/MS analysis on fasting plasma samples. The analysis was conducted on 50 metabolites, including 20 amino acids and 30 AcylCs, after injecting of a 5 μL sample. The mobile phase consisted of a mixture of 75% acetonitrile aqueous solution. To process the data and quantify the metabolites, the researchers employed the MultiQuant software (ABI Sciex) and used ratios of the signals of the metabolites relative to the isotopes (as internal standards) for calibration and calculation of analyte concentrations. For a detailed description of the analytical procedures, readers can refer to reference [16].

### 2.3 Data Analysis and Preprocessing

Two methods of dropping and imputation were used to handle missing values. Missing values in HbA1c and glucose levels were dropped from the data frame. Missing values among amino acids and AcylCs were imputed on the basis of the mean of each value.

The diabetes diagnosis and treatment standards of the American Diabetes Association (ADA) were used to classify participants into the prediabetic, diabetic and nondiabetic (healthy) groups. Furthermore, the diabetic group was stratified on the basis of glycaemic control into two groups. As recommended by the ADA, good glycaemic was defined on the basis of HbA1c target < 7% (GGC). The HbA1c level greater than 8% was defined as PGC [17].

NearestNeighbors was used as a sampling method to match controls and cases in each group on the basis of age, sex and BMI. The data were first normalised using the StandardScaler technique, and the number of neighbours (*k*) was selected as 1. The normal distribution of all numerical features was checked by the Shapiro–Wilk test [18]. The Mann–Whitney (independent samples) test was used as a non‐parametric test to check statistically significant features using false discovery rate (FDR) adjusted *p* < 0.05.

### 2.4 Metabolite Fold Change

Amino acids and AcylC values were normalised between 0 and 1 on the basis of the minimum and maximum of each data point. Each group of prediabetes, diabetes, GGC, PGC and GGC–PGC was classified into binary classes of 0 and 1 (0 = desirable and 1 = undesirable, except for the GGC–PGC group in which 0 and 1 were considered as the GGC and PGC groups, respectively). On the basis of the mean of each binary class in mentioned groups, the Log2 factor change (Log2FC) was calculated. Log2FC and −log10 MW *p*‐values were used to show each metabolite's fold changes as volcano plots.

### 2.5 Correlation Coefficients

Pearson's correlation coefficient [19] was used to check the correlation of both glucose and HbAc1 levels as independent factors with both binary classes (desirable/undesirable) in each group of prediabetes, diabetes, GGC, PGC and GGC–PGC.

The ComplexHeatmap [20] package in R was used to visualise correlation coefficients and *p*‐value heatmaps.

## 3 Results

**FIGURE 1:**
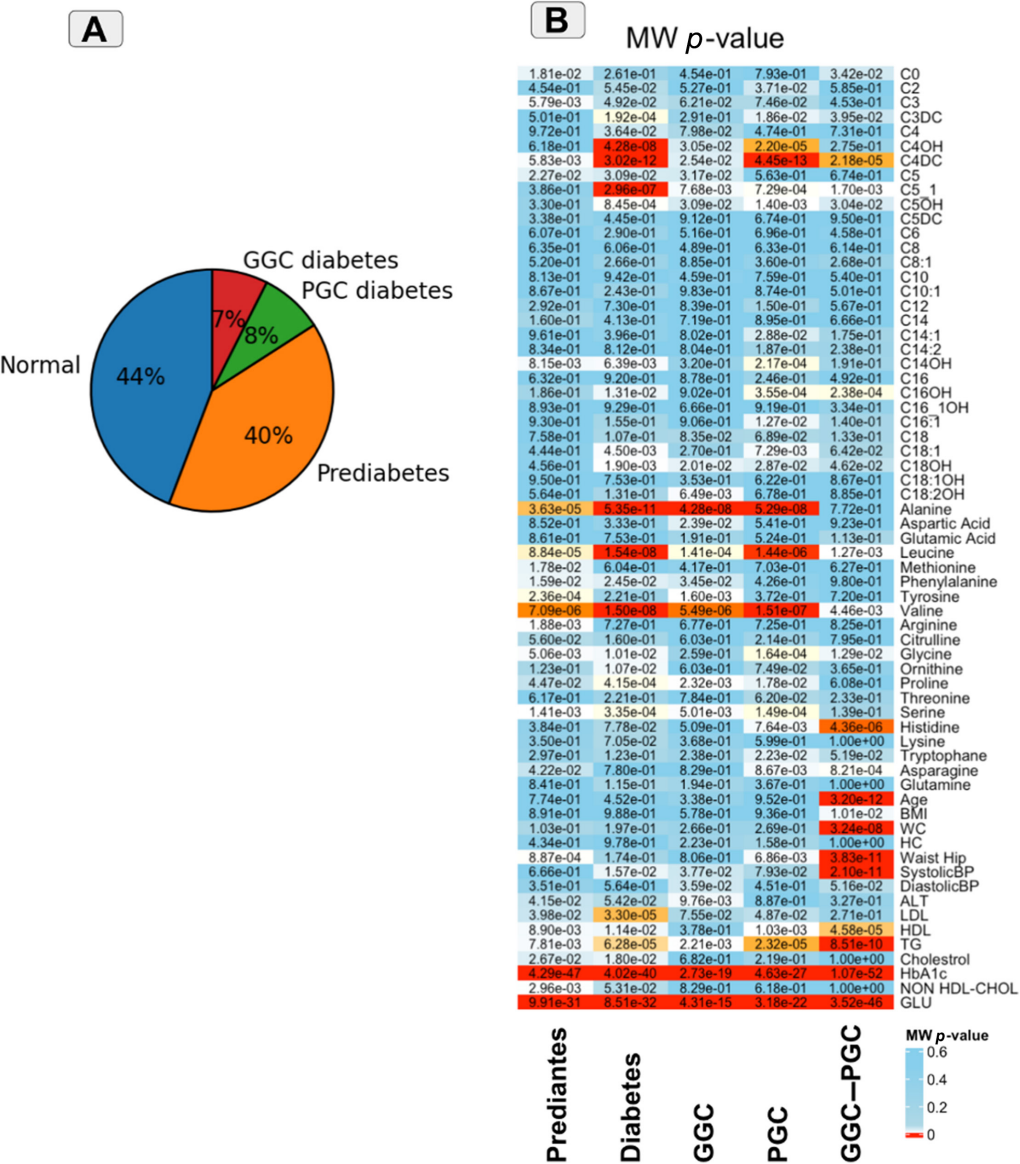
(A) Different groups of participants. (B) Heatmap of Mann–Whitney *p*‐values for all studied features in the prediabetes, diabetes, GGC, PGC and GGC–PGC groups on the basis of desirable (control) and undesirable (case) categories.

After dropping missing values, out of 1092 subjects, there were 485 normal, 433 prediabetes, 81 GGC and 93 PGC cases (Figure 1A). Table 1 shows the basic characteristics of different groups in this study.

**TABLE 1:** Study participants' basic characteristics are classified into different prediabetes (PD) and diabetes (DD) classes, including the good (GGC) and poor glycaemic control (PGC) groups.

Variables	PD N^b^ (*n* = 240)	PD A (*n* = 224)	*p*‐value^c^	DD N (*N* = 139)	DD A (*N* = 165)	*p*‐value	GGC A (*N* = 72)	*p*‐value	PGC A (*N* = 81)	*p*‐value	GP N (*N* = 90)	GP A (*N* = 79)	*p*‐value
Gender (*n*)^a^													
Female	251	228	ns	479	90	ns	45	ns	45	ns	45	45	ns
Male	219	196		415	79		34		45		45	34	
Area (*n*)													
Rural	151	157	ns	308	40	ns	23	ns	17	ns	17	23	ns
Urban	319	267		586	129		56		73		73	56	
Years of education (*n*)													
0	99	104	ns	203	56	ns	22	ns	34	ns	34	22	ns
1–6	158	152		310	60		30		30		30	30	
7–12	140	122		262	42		23		19		19	23	
> 12	73	46		119	11		4		7		7	4	
Smoking (*n*)													
No	401	361	ns	762	147	ns	68	ns	79	ns	79	68	ns
Yes	69	63		132	22		11		11		11	11	
Marriage (*n*)													
Single	11	10	ns	21	0	ns	0	ns	0	ns	0	0	ns
Married	422	361		783	147		71		76		76	71	
Divorced	6	8		14	2		1		1		1	1	
Widow	31	45		76	20		7		13		13	7	
BP_treatement (*n*)													
No	425	345	ns	770	113	ns	55	ns	58	ns	58	55	ns
Yes	45	79		124	56		24		32		32	24	
Oral treatment													
No	470	424	ns	894	84	ns	43	ns	41	ns	41	43	ns
Yes	0	0		0	85		36		49		49	36	
lipid_treat_now													
No	454	394	ns	848	124	ns	61	ns	63	ns	63	61	ns
Yes	16	30		46	45		18		27		27	18	
Insulin													
No	470	424	ns	894	153	ns	74	ns	79	ns	79	74	ns
Yes	0	0		0	16		5		11		11	5	
Age (year)	53.68 ± 9.78	53.58 ± 9.83	ns	53.46 ± 9.98	59.37 ± 10.09	ns	59.00 ± 10.53	ns	58.44 ± 9.44	ns	59.54 ± 10.60	59.21 ± 9.69	ns
BMI (kg/m^2^)	27.90 ± 4.70	27.96 ± 4.77	ns	27.66 ± 5.07	29.09 ± 4.89	ns	29.59 ± 4.55	ns	28.18 ± 4.83	ns	29.68 ± 4.66	28.57 ± 5.05	ns
< 18.5	11	14		25	1		0		1		1	0	
18.5–24.9	147	90		237	35		13		22		22	13	
25–29	204	163		367	64		29		35		35	29	
≥ 30	108	157		265	69		37		32		32	37	
WC (cm)	93.76 ± 12.69	95.97 ± 10.69	ns	98.93 ± 11.36	99.64 ± 13.11	ns	100.04 ± 11.68	ns	98.61 ± 14.51	ns	100.14 ± 11.57	99.16 ± 14.20	ns
HC (cm)	103.12 ± 11.22	102.49 ± 11.30	ns	104.14 ± 10.06	104.07 ± 10.03	ns	106.07 ± 9.52	ns	101.81 ± 10.20	ns	106.15 ± 9.63	102.08 ± 9.93	**
Waist/Hip (cm)	0.91 ± 0.09	0.94 ± 0.09	**	0.95 ± 0.08	0.96 ± 0.10	ns	0.94 ± 0.10	ns	0.97 ± 0.11	**	0.94 ± 0.09	0.97 ± 0.11	*
Systolic BP (mmHg)	130.20 ± 19.78	130.45 ± 19.52	ns	135.09 ± 23.42	140.39 ± 21.34	*	138.01 ± 19.81	ns	141.09 ± 22.88	ns	137.71 ± 19.61	142.16 ± 22.70	ns
Diastolic BP (mmHg)	80.61 ± 11.58	81.36 ± 11.30	ns	81.36 ± 12.22	82.98 ± 12.81	ns	83.76 ± 13.29	*	82.95 ± 12.96	ns	83.04 ± 13.13	82.57 ± 12.55	ns
Serum Cr (mg/dL)	0.87 ± 0.20	0.86 ± 0.19	ns	0.86 ± 0.20	0.88 ± 0.27	*	0.91 ± 0.21	ns	0.86 ± 0.31	ns	0.67 ± 0.01	0.59 ± 0.05	****
BUN (mmol/L)	15.20 ± 4.24	15.80 ± 4.10	ns	15.49 ± 4.233	16.86 ± 5.1	*	16.84 ± 5.48	ns	16.87 ± 4.71	ns	13.26 ± 3.67	13.06 ± 3.12	ns
Alb/Cr (mg/g)	12.33 ± 58.90	21.05 ± 75.42	*	16.44 ± 67.18	39.18 ± 132.86	****	21.88 ± 66.74	ns	53.88 ± 169.05	****	12.19 ± 26.83	22.23 ± 85.00	ns
GFR (mL/min/1.73 m^2^)	89.55 ± 15.82	86.86 ± 15.39	ns	88.32 ± 15.66	83.76 ± 18.87	****	79.74 ± 17.33	ns	87.16 ± 19.54	**	101.28 ± 6.91	105.79 ± 7.99	***
Uric Acid (mg/dL)	6.07 ± 1.50	5.17 ± 1.21	****	5.35 ± 1.40	4.49 ± 1.12	****	4.73 ± 1.16	****	4.32 ± 1.04	****	4.69 ± 1.18	4.33 ± 1.03	*
ALP (U/L)	80.32 ± 23.02	77.20 ± 20.98	ns	82.30 ± 24.04	78.72 ± 25.60	ns	77.07 ± 25.86	ns	80.54 ± 25.23	ns	75.62 ± 25.81	80.28 ± 25.43	ns
AST (U/L)	26.93 ± 9.26	27.91 ± 11.31	ns	27.82 ± 12.91	25.42 ± 10.31	*	27.42 ± 12.55	ns	23.40 ± 7.05	*	27.46 ± 12.65	23.53 ± 7.21	ns
ALT (U/L)	19.63 ± 9.34	22.62 ± 12.76	*	20.23 ± 9.13	22.80 ± 12.40	*	24.91 ± 15.10	*	21.08 ± 9.70	ns	24.75 ± 14.72	21.09 ± 9.39	ns
LDLc (mg/dL)	101.10 ± 29.50	106.12 ± 29.43	ns	107.21 ± 25.93	93.06 ± 33.56	****	95.56 ± 31.60	ns	89.75 ± 35.24	*	96.06 ± 31.85	91.22 ± 34.67	ns
HDLc (mg/dL)	42.58 ± 11.54	40.28 ± 11.34	*	40.88 ± 10.28	38.46 ± 11.21	*	40.05 ± 12.29	ns	37.11 ± 10.60	**	39.87 ± 11.86	37.04 ± 10.35	ns
TG (mg/dL)	130.08 ± 89.63	145.66 ± 88.64	*	133.13 ± 70.37	176.51 ± 169.81	***	190.08 ± 240.92	**	167.36 ± 83.77	***	186.60 ± 230.38	167.87 ± 80.43	ns
Chol (mg/dL)	169.16 ± 34.98	175.47 ± 34.55	*	173.92 ± 32.23	165.55 ± 44.18	*	170.37 ± 45.13	ns	160.62 ± 44.17	ns	170.30 ± 44.43	162.09 ± 43.13	ns
HbA1c (%)	5.28 ± 0.25	5.74 ± 0.33	****	5.62 ± 0.37	7.65 ± 1.82	****	6.27 ± 0.48	****	8.91 ± 1.72	****	6.27 ± 0.47	8.88 ± 1.68	****
NHC (mg/dL)	126.58 ± 34.22	135.19 ± 34.25	**	133.04 ± 30.51	126.98 ± 43.04	ns	130.32 ± 45.85	ns	123.30 ± 41.46	ns	130.43 ± 45.00	124.87 ± 40.71	ns
GLU (mg/dL)	87.09 ± 9.07	98.58 ± 11.66	****	94.76 ± 10.08	154.86 ± 63.13	****	119.52 ± 24.05	****	188.43 ± 71.63	****	118.90 ± 24.65	186.50 ± 69.01	****

^a^ Continuous variables are presented as mean ± SD, and categorical variables are presented as the number of each variable.

^b^ Abbreviations: A, abnormal; Alb/Cr, albumin‐creatinine ratio; ALP, alkaline phosphatase; ALT, alanine transaminase; AST, aspartate aminotransferase; BMI, body mass index; BP, blood pressure; BUN, Blood urea nitrogen; Chol, cholesterol; DD, diabetes; GF, glomerular filtration rate; GGC, good glycemic control; GLU, glucose; GP, good glycemic control vs poor glycemic control; HbA1c, Hemoglobin A1c; HC, hip circumference; HDLc, High‐density lipoprotein cholesterol; LDLc, Low‐density lipoprotein cholesterol; N, normal; NHC, Non‐HDL cholesterol; ns, not significant; PD, prediabetes; PGC, poor glycemic control; Serum Cr, serum creatinine; TG, Triglycerides; WC, waist circumference.

^c^ The Chi‐square test was employed to analyze the significant associations between categorical variables. On the other hand, for all numerical features, the non‐parametric Mann–Whitney test (independent samples) was used. *p*‐Value annotation legend: *: 1.00e‐02 < *p* ≤ 5.00e‐02, **: 1.00e‐03 < *p* ≤ 1.00e‐02, ***: 1.00e‐04 < *p* ≤ 1.00e‐03, ****: *p* ≤ 1.00e‐04.

### 3.1 Metabolite Differences Between Studied Groups

Metabolites with *p* < 0.01 were considered and reported as statistically significant (Figure 1B). The analysis showed that C3 and arginine were the only metabolites that increased explicitly in the undesirable (abnormal) prediabetes group. C5:1 also did not show any differences in the prediabetes group between control and case, but in other groups, it had a significant difference between controls and cases.

C18:2OH also statistically decreased just in GGC but not even in the PGC groups. On the contrary, C16OH concentrations were statistically different in the PGC diabetes group, and differences between the GGC and PGC groups were confirmed by showing the differences in the GGC–PGC group.

Alanine, leucine, valine and serine also showed significant changes in all prediabetes, diabetes, GGC and PGC groups. However, the concentrations of histidine and asparagine were statistically significant in only PGC diabetes groups, although these differences were also evident in the GGC–PGC group.

C4DC showed statistically significant changes in all groups except the GGC diabetes group. This difference between the GGC and PGC diabetes groups regarding C4DC also was confirmed by showing statistically significant changes in C4DC in the GGC–PGC group.

### 3.2 Fold Change

**FIGURE 2:**
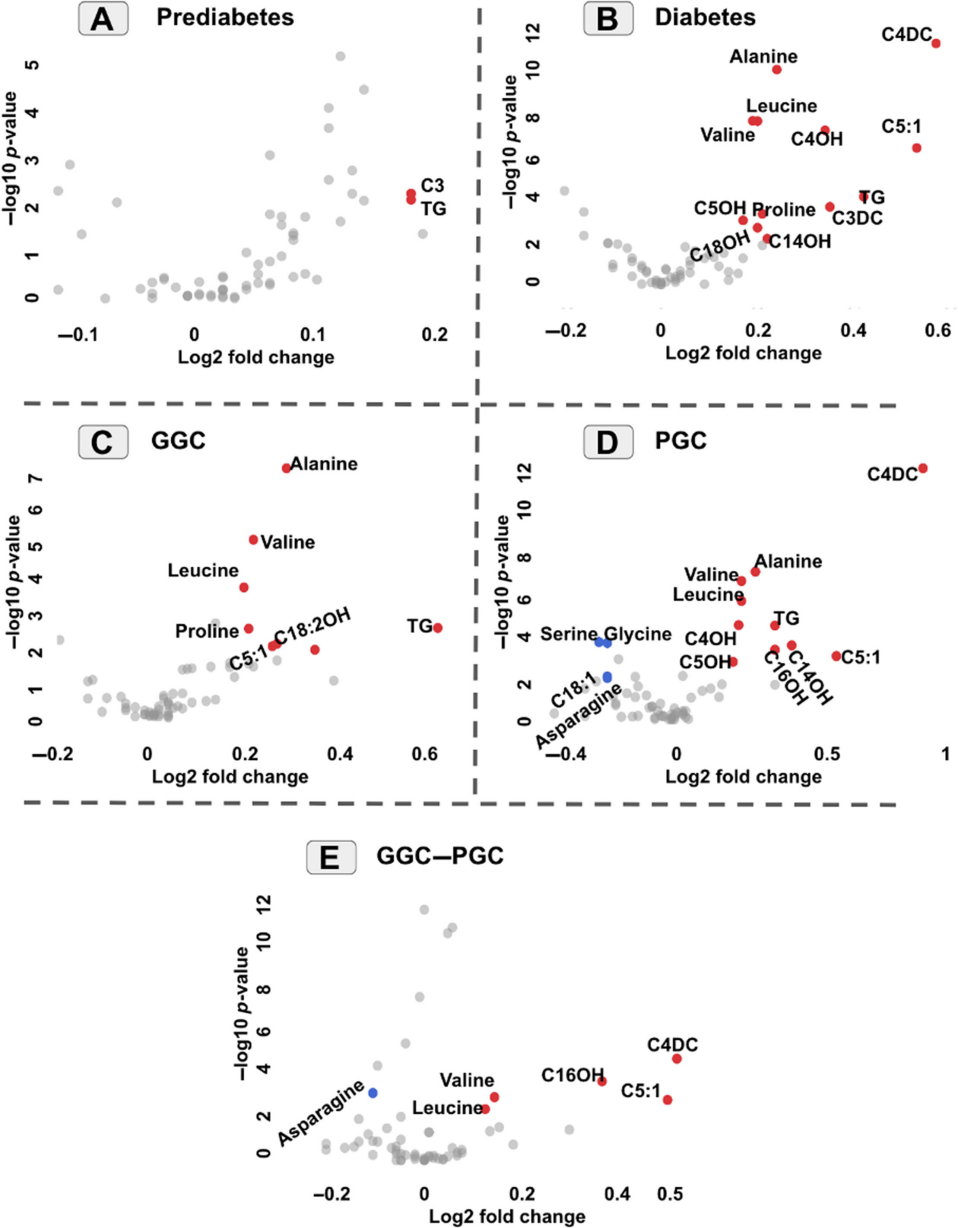
Fold change analysis for prediabetes (A), diabetes (B), GGC (C), PGC (D) and GGC–PGC (E).

Fold changes for all studied groups are illustrated in Figure 2. Metabolites with two conditions were reported as significant fold change: (1) −log10 *p*‐value metabolites must be > 2 (*p* < 0.01), and (2) fold change must be > 1.1 (Log2FC > 0.15) or < 0.9 (Log2FC < −0.15). In Figure 2, increased and decreased fold changes are plotted as red and blue dots, respectively.

In the prediabetes group (Figure 2A), C3 concentration was reported as increased fold change with a fold change score of 1.13.

Alanine, leucine, valine and proline were the only amino acids that showed increased fold changes in the diabetes group (Figure 2B). AcylCs, including C4DC, C5:1, C4OH, C5OH, C14OH, C18OH and C3DC, were defined as increased fold change in the diabetes group. Same as the diabetes group, in the GGC group, alanine, leucine and valine concentrations had increased fold change as compared to the control group (Figure 2C). C5:1 and C18:2OH also reported increased fold change.

Interestingly, in the PGC group, serine, glycine, asparagine and C18:1 showed decreased fold change (Figure 2D). On the contrary, alanine, leucine, valine, C5:1, C4OH, C5OH, C14OH and C16OH had increased fold change as compared to the control group.

In the GGC–PGC group (Figure 2E), which was defined as a way to compare the GGC and PGC groups, asparagine in the PGC group showed decreased fold change as compared to the GGC group. However, valine, leucine, C5:1, C16OH and C4DC had increased fold change in the PGC group.

### 3.3 Pearson Correlation

**FIGURE 3:**
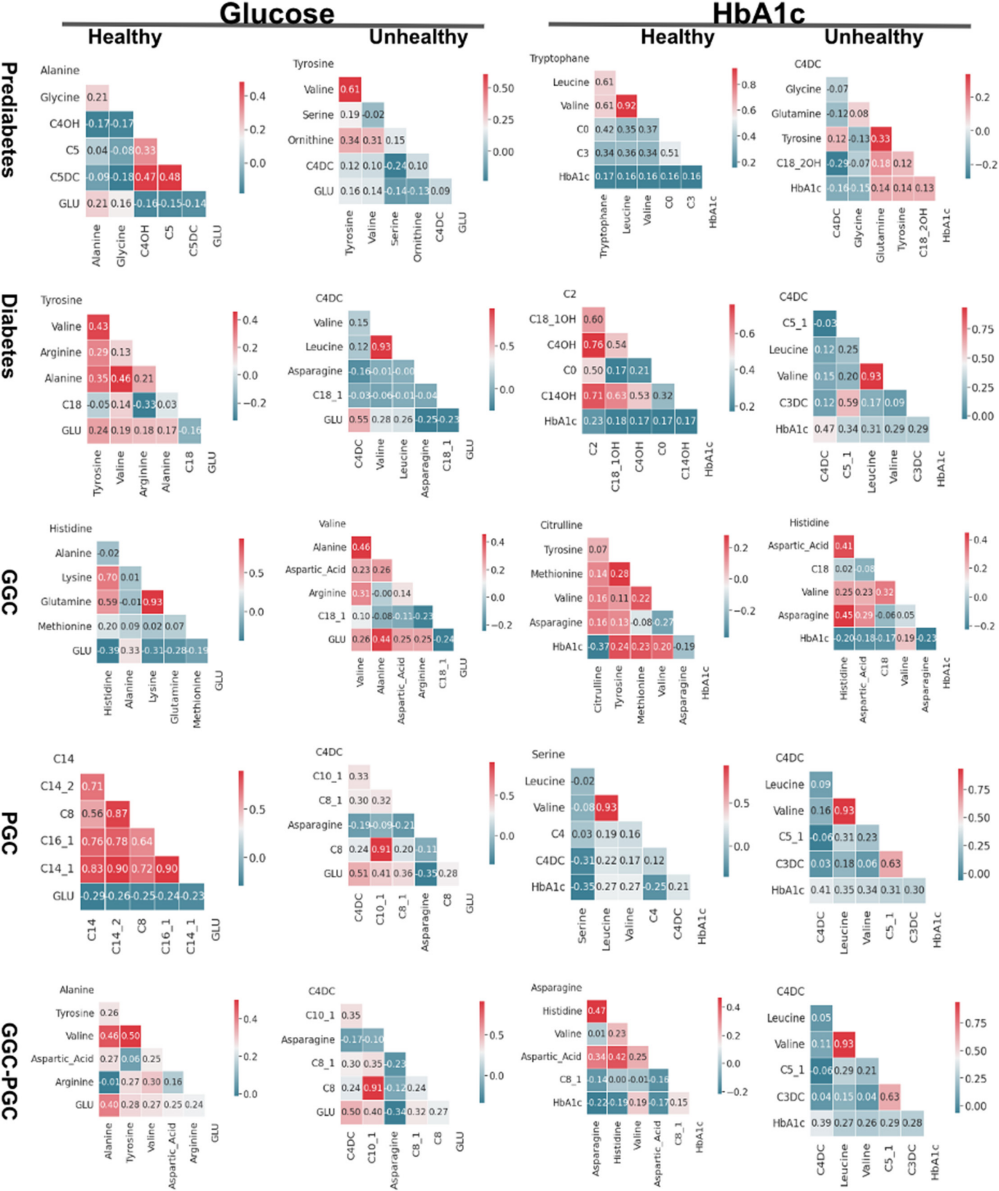
Pearson correlation coefficient of glucose and HbA1c with metabolites in the prediabetes, diabetes, GGC, PGC and GGC–PGC groups. Healthy and unhealthy stand for control and case in each group (except for GGC–PGC, which refers to controlled and uncontrolled diabetes).

In this study, Pearson's correlation coefficients were applied to show the strength of the linear relationship of glucose and HbA1c between metabolites in both desirable and undesirable models in the prediabetes, diabetes, GGC, PGC and GGC–PGC groups (Figure 3). In both the desirable and undesirable prediabetes groups, glucose and HbA1c did not show any moderate or powerful (> 0.3) relationship with metabolites.

In the diabetes group, glucose and HbA1c scores in people with desirable (healthy) scores did not show a correlation with metabolites. On the contrary, glucose and HbA1c had a positive correlation with C4DC and C5:1, leucine and C4DC in people with diabetes with undesirable (unhealthy) scores, respectively.

As shown in Figure 3 for the GGC group, HbA1c only correlated with citrulline in people with desirable values. Alanine in both desirable and undesirable classes showed a positive correlation with glucose. Also, histidine and lysine positively correlated with glucose in the desirable GGC group.

Serine in people with undesirable values in the PGC group had a positive correlation with HbA1c but not with glucose. However, in the PGC group, both glucose (with C4DC, C10:1, C8:1 and asparagine) and HbA1c (with C4DC, leucine, valine, C5:1 and C3DC) showed a weak‐to‐moderate correlation with metabolites.

## 4 Discussion

The global prevalence of Type 2 diabetes mellitus (T2DM) has attracted wide attention because of its financial burden on healthcare systems [21]. Although the diagnosis of diabetes or prediabetes can be accomplished by a simple measurement of blood glucose, short‐term glycaemic changes alone are not accurate and may generate false positive results [8]. Therefore, identifying additional biomarkers is needed for early prevention, management and treatment of diabetes [22]. This study comprehensively examined plasma metabolites (amino acids and AcylCs) in the prediabetes and diabetes (GGC and PGC) groups using targeted LC–MS/MS metabolomics.

The novel finding in our article was that asparagine had different fold changes in the PGC and GGC groups. The studies in Tianjin Medical University found that abnormal asparagine and aspartate homeostasis contributed to an increased risk of T2DM, and the results were the same as ours [23]. It has been shown that the elevation of asparagine's level in the serum of the population has an inverse relationship with the progression of diabetes risk [24].

AcylCs are intermediate oxidative metabolites constructed from a fatty acid esterified to carnitine [25]. Fatty acid oxidation (FAO) mainly happens in mitochondria and involves repeated reactions that result in energy production [26]. Long‐chain fatty acids are first activated in the cytosol to fatty acyl‐CoAs. Because of the lack of acyl‐CoA transfer proteins, acyl‐CoAs are transported into the mitochondrion by the carnitine shuttle system. In mitochondria, multi‐step reactions are implemented to generate acetyl‐CoA, which provides energy by participating in the tricarboxylic acid cycle (TCA cycle) [27].

AcylC metabolism has been broadly examined regarding T2DM and insulin resistance in different populations [28, 29, 30, 31, 32]. However, we know little about the role of AcylCs in various stages of diabetes. This study showed that C3, C4DC, C5:1, C4OH, C5OH and C3DC as short‐chain AcylCs and C14OH, C16OH, C18OH and C18:2OH as hydroxylated long‐chain AcylCs were positively associated with diabetes risk. These findings are supported by Hosseinkhani et al., who show that short and hydroxylated long‐chain AcylCs increased in people with diabetes compared with controls in Iran's population [13]. We did not observe any significant associations between medium‐chain species and diabetes, like the study that was done in the Asian population [33]. Compared with other groups, only prediabetes showed increased C3 levels, which may be a significant predictive biomarker for prediabetes transition to diabetes. According to the Mai et al. study, there were significant differences in concentrations of C3 and C3DC + C4OH between the prediabetic conditions [29]. In the PGC versus GGC groups, C5:1, C16OH and C4DC AcylCs showed increased fold change. C4DC showed a positive correlation with glucose, and like C16OH augmented only in PGC.

Dicarboxylic species, including C4DC, are produced when β‐oxidation of long‐chain fatty acids is disturbed, and the compensatory path of ω‐oxidation is activated [34]. These species could promote the expression of genes and proteins related to oxidative stress [35]. In the study by Mihalik et al., a nearly doubled elevation in C4DC level was observed in T2DM compared with obese or lean participants that correlated with two indexes of PGC [30]. In another study, higher plasma and serum levels of specific amino acids were associated with a higher risk of T2DM [36]. C4DC may be a valuable biomarker of glucolipotoxicity in T2DM [37].

The accumulation of long‐chain species, such as C16‐OH, the initial products of β‐oxidation, is associated with insulin resistance [38, 39]. A German study reported higher concentrations of C16‐OH in participants with diabetes compared with those with normal glucose tolerance [29]. This finding is consistent with another report, which found that overall metabolite levels increased with an accumulation of C16‐OH–AcylCs in diabetes [33].

C3 and C5 AcylCs produced during the catabolism of BCAA were higher in obese and T2DM subjects compared with lean controls [40]. Also, the levels of C3 and C5‐I were significantly higher in the GDM (gestational diabetes mellitus) group and associated with increased GDM risk in early pregnancy [41]. The accumulation of these AcylCs, showing generalised dysfunction at the interface of FAO and the electron transport chain (ETC), could activate proinflammatory pathways and exacerbate insulin resistance [42].

## 5 Conclusion

In conclusion, our study provides valuable insights into the metabolic differences among normal, prediabetic and diabetic individuals, with a focus on glycaemic control status. Using tandem mass spectrometry analysis, we identified significant alterations in amino acids and AcylCs that distinguish prediabetes and diabetes from healthy individuals. These findings shed light on potential metabolic biomarkers associated with diabetes risk and progression.

## 6 Acylcarnitine Names

Free carnitine (C0), acetyl carnitine (C2), propionyl carnitine (C3), malonyl carnitine (C3‐DC), butyryl carnitine (C4), methylmalonyl‐/succinylcarnitine (C4‐DC), 3‐OH‐iso‐/butyryl carnitine (C4‐OH), isovalerylcarnitine (C5), tiglylcarnitine (C5:1), 3‐OH‐isovalerylcarnitine (C5‐OH), glutarylcarnitine (C5DC), hexanoyl carnitine (C6), octanoylcarnitine (C8), octenoylcarnitine (C8:1), decanoylcarnitine (C10), decenoylcarnitine (C10:1), dodecanoyl carnitine (C12), tetradecanoyl carnitine (C14), tetradecenoyl carnitine (C14:1), tetradecadienoyl carnitine (C14:2), 3‐OH‐tetradecanoylcarnitine (C14‐OH), hexadecanoyl carnitine (C16), 3‐OH‐hexadecanoylcarnitine (C16‐OH), 3‐OH‐hexadecenoylcarnitine (C16:1‐OH), hexadecenoyl carnitine (C16:1), octadecanoyl carnitine (C18), octadecenoyl carnitine (C18:1), 3‐OH‐octadecanoylcarnitine (C18‐OH), 3‐OH‐octadecenoylcarnitine (C18:1‐OH), octadecadienoyl carnitine (C18:2).

## Author Contributions

**Saad Ayyal Jabbar Al‐Rikabi:** Funding acquisition (equal); Investigation (equal); Methodology (equal); Project administration (equal); Validation (equal); Visualization (equal); Writing – original draft (equal); Writing – review and editing (equal). **Ali Etemadi:** Investigation; Methodology; Validation; Visualization; Writing; Software; Data analysis. **Maher Mohammed Morad:** Data curation (equal); Formal analysis (equal); Funding acquisition (equal); Software (equal); Supervision (equal); Validation (equal). **Azin Nowrouzi:** Conceptualization (equal); Data curation (equal); Formal analysis (equal); Funding acquisition (equal); Investigation (equal). **Ghodarollah Shayriyar Panahi:** Funding acquisition (equal); Investigation (equal); Methodology (equal); Project administration (equal); Validation (equal); Visualization (equal); Writing – original draft (equal); Writing – review and editing (equal). **Mozhgan Mondeali:** Conceptualization (equal); Data curation (equal); Resources (equal); Software (equal). **Mahsa Toorani‐ghazvini:** Conceptualization (equal); Data curation (equal); Resources (equal); Software (equal). **Ensieh Nasli‐Esfahani:** Validation; Visualization; Writing. **Farideh Razi:** Conceptualization (equal); Data curation (equal); Formal analysis (equal); Resources (equal); Software (equal); Supervision (equal). **Fatemeh Bandarian:** Resources (equal); Software (equal); Supervision (equal); Validation (equal); Visualization (equal).

## Ethics Statement

The study protocol was approved by the Ethics Committee of Endocrinology and Metabolism Clinical Sciences Institute, Tehran University of Medical Sciences (IR.TUMS.EMRI.REC. 1395.00141).

## Consent

The purpose of the study was explained to the participants, and written informed consent was obtained from all participants.

## Conflicts of Interest

The authors declare that there are no conflicts of interest regarding the publication of this study.

## Data Availability

The data sets generated during and/or analysed during the current study are available from the corresponding author on reasonable request.
